# Rapid Design, Microstructures, and Properties of Low-Cost Co-Free Al-Cr-Fe-Ni Eutectic Medium Entropy Alloys

**DOI:** 10.3390/ma16010056

**Published:** 2022-12-21

**Authors:** Jiongpei Yuan, Yujing Yang, Shougang Duan, Yong Dong, Chuanqiang Li, Zhengrong Zhang

**Affiliations:** School of Materials and Energy, Guangdong University of Technology, Guangzhou 510006, China

**Keywords:** alloy design, eutectic medium entropy alloys, microstructure, properties

## Abstract

In this study, we establish a mathematical rule for accelerating the prediction of low-cost Co-free AlCr_a_Fe_b_Ni_c_ FCC/B2-structured eutectic medium entropy alloys (EMEAs). The mathematical formulas are c ≥ 1.0, 4.38a + 4.28b + 3.97c ≈ 20.55, and c − a ˃ 1.0. With this rule, we successfully predict the AlCr_1.18_FeNi_2.8_ and AlCrFe_1.46_Ni_2.5_ eutectic alloys and verify their eutectic morphology by experiments. Both the AlCr_1.18_FeNi_2.8_ and AlCrFe_1.46_Ni_2.5_ EHEAs exhibit excellent compressive mechanical properties, with yield stress higher than 500 MPa, compressive fracture strength higher than 2450 MPa, and fracture strain > 40%, which can be attributed to their unique lamellar microstructure. Moreover, both alloys exhibit good corrosion resistance in 3.5 wt.% NaCl solution. Among them, the AlCr_1.18_FeNi_2.8_ EHEA exhibits better corrosion resistance due to the higher content of the FCC phase.

## 1. Introduction

Recently, high-entropy alloys (HEAs) have received widespread attention due to their novel design concept and excellent performance [1,2]. Generally, HEAs consist of five or more major elements where the content of each element ranges from 5 to 35% [2]. Although there are multiple principal elements in HEAs, they tend to form simple structures such as face-centered-cubic (FCC), body-centered-cubic (BCC), and a mixture of FCC and BCC rather than complex intermetallic compounds. Nevertheless, not all HEAs have excellent comprehensive performance [3,4,5,6]. Single-phase with FCC structure HEAs have high plasticity but low strength, while the BCC HEAs have high strength and poor plasticity. For example, the elongation of CoCrFeMnNi HEA with FCC structure can reach ~50%, but the strength is only ~410 MPa [7]. While the compressive strength of the BCC-structured AlCoCrFeNi HEA reaches 2864.9 MPa, the compression ratio is only 22.7% [8]. This phenomenon is referred to as the strength ductility trade-off [9]. In addition, single-phase HEAs also face the problem of poor casting fluidity [10]. To address these shortcomings, Lu et al. proposed a novel alloy with fine dual-phase lamellar microstructure and almost no casting defects based on the combined advantages of eutectic alloys and HEAs, named eutectic high entropy alloys (EHEAs) [11]. The elongation of AlCoCrFeNi_2.1_ EHEA reaches 17%, and the tensile strength is more than 1 GPa. The available studies have shown that the EHEAs also have excellent phase stability even at high temperatures, which greatly improves their industrial application value [12,13].

To date, more and more EHEAs have been developed, such as CrFeNi_2_Al EHEA with FCC/B2-structured [14], Al_0.75_CrFeNi EHEA with BCC/B2-structured EHEAs [15], CoCrFeNiNb_x_ EHEAs with FCC/intermetallic compound (IMC)-structured [16]. Among the reported EHEAs, the FCC/B2 dual-phase alloys have received the most attention due to their excellent properties and phase stability both under low and high temperatures. Therefore, it is urgent to develop more FCC/B2-structured EHEAs. Although many methods have been proposed for designing EHEAs, most of them have low success rates and are time-consuming [17,18,19,20]. For example, the traditional trial-and-error method consumes a lot of time and has a low success rate [17]. Therefore, the rapid design of FCC/B2-structured EHEAs is still a great challenge. Most of the reported FCC/B2-structured EHEAs contain expensive Co element, which means their industrial applications have been limited [21,22]. Furthermore, it was found that removing the expensive Co element does not reduce the strength and plasticity of the EHEAs [23]. In previous work, we have successfully designed eight FCC/B2-type near-EHEAs in the Al-Co-Cr-Fe-Ni alloy system by a simple empirical mathematical rule, which means that this rule has great potential in the composition design of EHEAs [24]. Despite the AlCo_a_Cr_b_Fe_c_Ni_d_ EHEAs exhibiting excellent mechanical properties and casting fluidity, the expensive Co element greatly limits its industrial application. In this study, we have extended this rule to the Al-Cr-Fe-Ni alloy system and successfully designed and prepared two eutectic medium entropy alloys (EMEAs). Additionally, the microstructure, compression properties, and corrosion resistance were investigated.

## 2. Composition Design and Experiment

### 2.1. Composition Design

**Figure 1:**
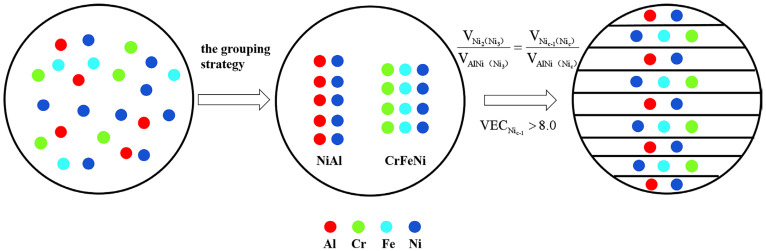
Schematic diagram of alloy design idea.

Based on the previous study of Lu et al. [19], a negative mixing enthalpy for the elements in the EHEAs system tends to form intermetallic compounds, while that close to zero tends to form a solid solution [25]. Based on this, the AlCrFeNi_3_ (denoted as Ni_3_) EMEA can be divided into the intermetallic compound NiAl phase group and solid solution CrFeNi_2_ phase group. Moreover, Guo et al. investigated the effect of valence electron concentration (VEC) on the stability of FCC and BCC solid solutions in HEAs. Based on the VEC criterion [26], the CrFeNi_2_ phase has a stable FCC structure, while the NiAl phase exhibits an ordered BCC structure. Similarly, we reasonably predict that the AlCr_a_Fe_b_Ni_c_ EMEAs were divided into NiAl intermetallic phase and the Cr_a_Fe_b_Ni_c−1_ (Ni_c−1_) solid solution phase. To guarantee the FCC structure of Ni_c−1_ phase, the Ni_c−1_ FCC phase also should satisfy the empirical rule VEC_Nic−1_ > 8.0. The VEC_Nic−1_ can be obtained from Equation (1), and the physicochemical properties of the elements used in the experiment are listed in Table 1. To ensure the existence of the mixture eutectic structure of the NiAl intermetallic phase and Cr_a_Fe_b_Ni_c−1_ solid solution phase, the eutectic structure can be achieved by mixing the two phases in a certain proportion, i.e., the volume ratio of the B2 phase (V_B2_) and FCC phase (V_FCC_) is approximately a constant value. The AlCrFeNi_3_ EMEA is selected to obtain the ideal V_B2_/V_FCC_ value, which is calculated to be 0.602 according to Equation (2). Figure 1 shows the design idea of EMEAs in the Al-Cr-Fe-Ni system. The three basic conditions, i.e., c ≥ 1.0, VEC_Nic−1_ > 8.0, and V_NiAl_/V_Nic−1_ = 0.602, can be adopted to obtain mathematical rules for predicting and designing FCC/B2 AlCr_a_Fe_b_Ni_c_ near EMEAs. By further calculation, the mathematical formulas are c ≥ 1.0, 4.38a + 4.28b + 3.97c ≈ 20.55, and c − a ˃ 1.0. Moreover, we also designed and prepared two alloys in the Al-Cr-Fe-Ni alloy system, i.e., AlCr_1.18_FeNi_2.8_ and AlCrFe_1.46_Ni_2.5_ alloys to verify the effectiveness of the rules.

(1) $$\mathrm{VEC}=\sum_{i=1}^{n}C_{i}{\left(\mathrm{VEC}\right)}_{i}$$

(2) $$V=\sum_{i=1}^{n}\frac{C_{i}M_{i}}{\rho_{i}}$$

where n is the number of the components in an alloy system; C_i_ is the atomic percentages of the ith element; (VEC)_i_ is the VEC for individual element; M_i_ is the relative atomic mass of the ith element; ρ_i_ is the density of the ith element.

### 2.2. Experiment

#### 2.2.1. Preparation

The button-like ingots (approximately 25 g) of the AlCr_1.18_FeNi_2.8_ and AlCrFe_1.46_Ni_2.5_ alloys were prepared by vacuum arc melting under a pure argon atmosphere. The purity of the raw materials Al, Cr, Fe, and Ni are higher than 99.9 wt%. All the experimental alloys were remelted at least five times for chemical homogeneity. The ingots were directly solidified in a water-cooled copper hearth with a diameter of 18 mm and a thickness of 12 mm.

#### 2.2.2. Microstructure and Composition Analysis

The samples with a gauge size of ∅10 mm × 10 mm were cut from the button-like ingots to observe microstructure morphology. After grinding, polishing, and etching with aqua regia, the samples were observed under an optical microscope (DMi8C, Leica, Germany). The phase identification was characterized by a Bruker D8 ADVANCE X-ray diffractometer (XRD, D8 ADVANCE, Bruker, Bremen, Germany) with a Cu target operated at 40 kV and 40 mA. The high magnification microstructure and phase composition were characterized by transmission electron microscopy (TEM, Talos F200S, FEI, Hillsboro, OR, USA) equipped with energy dispersive spectroscopy (EDS). The TEM specimens were prepared by traditional cutting, mechanical grinding, polishing, and twin-jet electro-polishing techniques (electrolyte: 95% ethanol + 5% perchloric acid, in vol%).

#### 2.2.3. Compressive Test

The cylindrical compressive samples with a diameter of 4 mm and a height of 8 mm were taken from casting ingots. Before the compression tests, the samples were ground to 1500 grit with SiC sandpaper to remove cut marks. Compressive tests were performed on an AG-X testing system (Shimadzu, Kyoto, Japan) at an initial strain rate of 1 × 10^−3^ s^−1^. Three compression tests were measured to obtain reproducible data.

#### 2.2.4. Electrochemical Measurements

Electrochemical tests were performed to assess the corrosion resistance of the AlCr_1.18_FeNi_2.8_ and AlCrFe_1.46_Ni_2.5_ alloys. In potentiodynamic polarization tests, a platinum electrode served as the counter electrode, and the saturated calomel electrode was the reference electrode, while the samples were the working electrode in a three-electrode cell. The alloys used as work electrodes were embedded in epoxy resin with an exposed working area of 0.1256 cm^2^. The test solution was 3.5 wt.% NaCl solution. The electrochemical measurements were performed at room temperature (25 °C). Prior to the tests, the samples were ground with SiC sandpaper up to 2000 grit, then grounded and polished. The working electrodes were initially reduced potentiostatically at −1 V for 15 min to remove the oxide film formed in the air, and then the open circuit potential (OCP) 3600 s was monitored to obtain the steady-state potential. The potentiodynamic-polarization test was carried out at a scan rate of 1 mV/min with an initial potential of −0.8 V vs. OCP until the current density reached a maximum of 1 mA/cm^2^. The electrochemical impedance spectroscopy (EIS) tests were performed at the OCP with a sinusoidal potential amplitude of 10 mV, running from 105 to 10^−2^ Hz. To confirm data reproducibility, the polarization tests were repeated at least three times.

## 3. Results and Discussion

### 3.1. Microstructure

**Figure 2:**
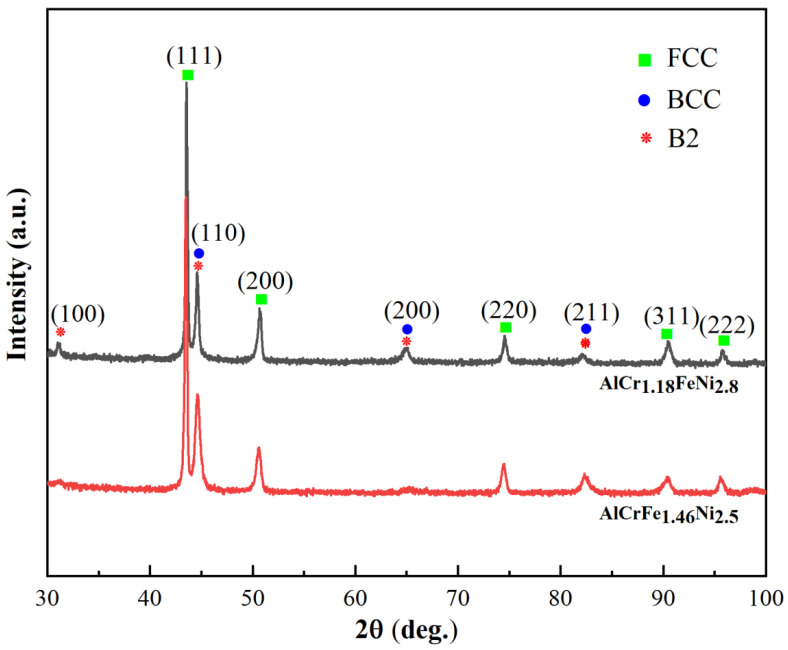
XRD patterns of the AlCr_1.18_FeNi_2.8_ and AlCrFe_1.46_Ni_2.5_ alloys.

**Figure 3:**
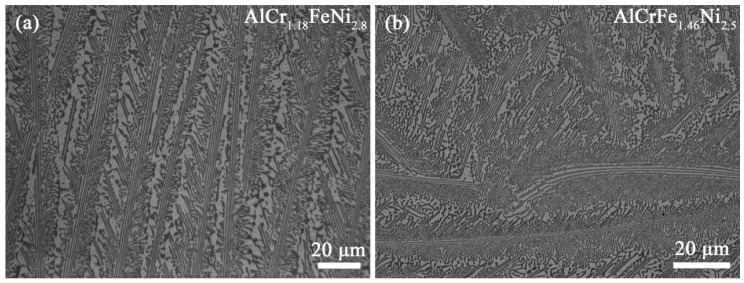
Optical micrographs of as-cast AlCr_a_Fe_b_Ni_c_ alloys. (**a**) AlCr_1.18_FeNi_2.8_ alloy; (**b**) AlCrFe_1.46_Ni_2.5_ alloy.

Figure 2 shows the XRD patterns of AlCr_1.18_FeNi_2.8_ and AlCrFe_1.46_Ni_2.5_ alloys. Both the two alloys show strong diffraction peaks of FCC and BCC phases. Moreover, a super-lattice peak around 31° implied the existence of an ordered BCC (B2) phase. The metallograph of the two alloys is shown in Figure 3. The two alloys exhibit a typical lamellar eutectic microstructure, which is similar to that of AlCrFeNi_3_ EMEA [25]. The phase compositions in the AlCr_1.18_FeNi_2.8_ and AlCrFe_1.46_Ni_2.5_ alloys are analyzed by TEM-EDS and shown in Table 2. The FCC phase is rich in Fe, Cr, and Ni elements but poor in Al elements, while the B2 phase is rich in Al and Ni elements but poor in Fe and Cr elements. In the process of calculating the phase volume ratio, we evaluate the phase volume ratio by processing several low-magnification metallographic pictures to calculate the area ratio of different phases. The V_B2_/V_FCC_ values for AlCr_1.18_FeNi_2.8_ and AlCrFe_1.46_Ni_2.5_ alloys are 0.605 and 0.679, respectively, which are close to the values of the mathematical rules established. Exhilaratingly, the existence of FCC and B2 phases verify the correctness of the grouping method of the component design.

**Figure 4:**
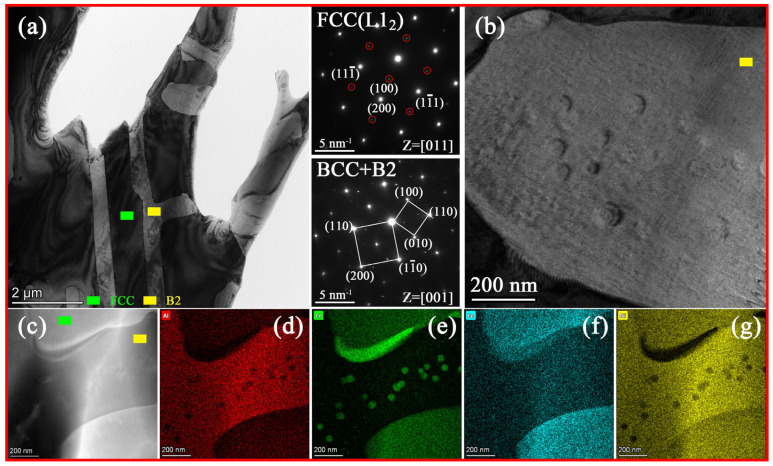
TEM analysis results of the AlCr_1.18_FeNi_2.8_. alloy. (**a**) Bright-field image; (**b**) High magnified image of the B2 phase; (**c**) HAADF–STEM image; (**d**–**g**) Elemental distribution EDS maps.

**Figure 5:**
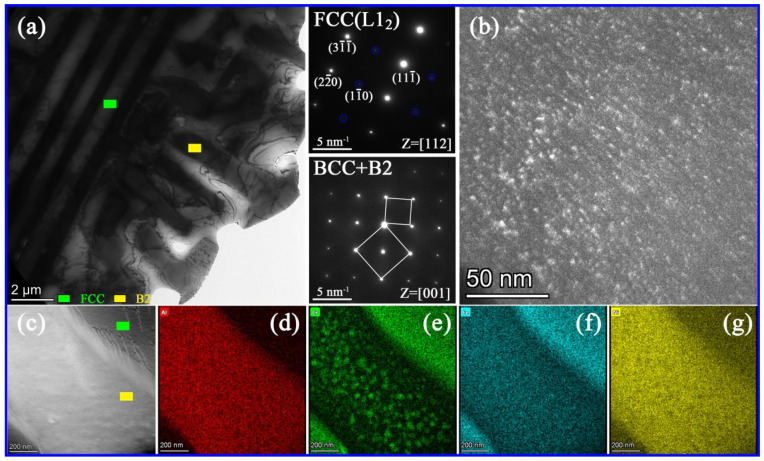
TEM analysis results of the AlCrFe_1.46_Ni_2.5_ alloy. (**a**) Bright-field image; (**b**) Dark field image of the FCC phase; (**c**) HAADF–STEM image; (**d**–**g**) Elemental distribution EDS maps.

Figure 4a and Figure 5a exhibit the bright-field TEM image of AlCr_1.18_FeNi_2.8_ and AlCrFe_1.46_Ni_2.5_ alloys, respectively. The two alloys suggest fine lamellar eutectic structures. The selected area diffraction patterns (SAEDs) show that in eutectic microstructure, both phases exhibit super-lattice reflections, confirming the existence of ordered FCC (L1_2_) structure (highlighted by green squares in Figure 4a and Figure 5a) and B2 structure (highlighted by yellow squares in Figure 4a and Figure 5a). Figure 4b displays a high-magnification bright-field TEM image of the B2 phase in AlCr_1.18_FeNi_2.8_ alloy and shows uniform distribution of spherical nanoparticles. As shown in Figure 4c–g, element segregation exists between the spherical nanoparticles and its B2 matrix, and the Cr element tends to segregate into the nanoparticles. Moreover, some block Cr-rich phase can be found between the FCC and B2 phase boundary; similar results were also reported in the other literature [14]. Figure 5b is a dark-field image along the (1–10) crystal plane showing the FCC phase contains a large amount of L1_2_ nanoprecipitates. Figure 5c–g shows the elemental distribution maps of AlCrFe_1.46_Ni_2.5_ alloy, indicating that FCC phases enrich Fe and Cr elements, while B2 phases enrich Al and Ni elements. The Cr-rich spherical nanoparticles are also found in the B2 phase of AlCrFe_1.46_Ni_2.5_ alloy. Factually, element segregation occurs between L1_2_ nanoprecipitate and FCC matrix, but it is difficult to distinguish due to the extremely small size of nanoprecipitates [27]. In Al-(Co)-Cr-Fe-Ni EHEAs system, it is often reported that the Cr-rich phase has BCC structure and is coherent with its B2 matrix, while the L1_2_ is an AlNi_3_-type γ′ phase and highly coherent with its FCC matrix [23,28].

### 3.2. Mechanical Properties

**Figure 6:**
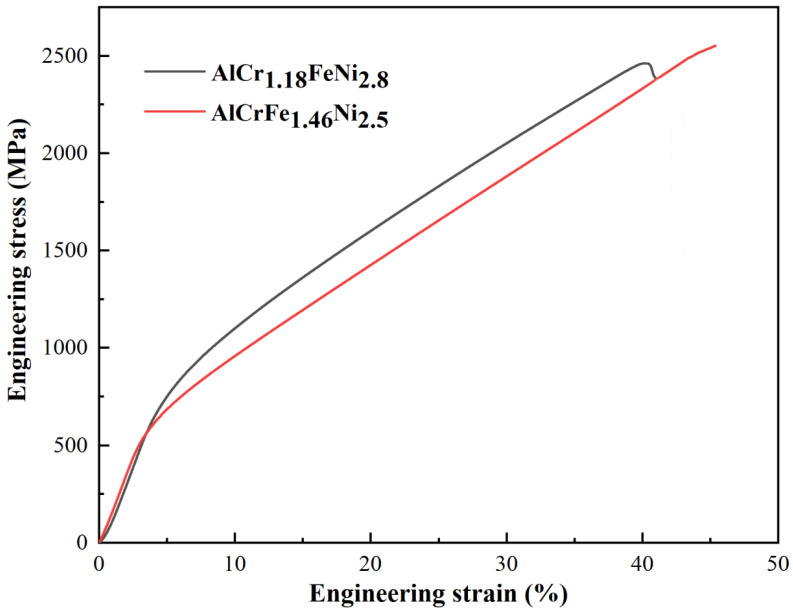
Engineering stress–strain curves of AlCr_1.18_FeNi_2.8_ and AlCrFe_1.46_Ni_2.5_ alloys at room temperature.

Figure 6 shows the compressive strain–stress curves of the AlCr_1.18_FeNi_2.8_ and AlCrFe_1.46_Ni_2.5_ alloys. The fracture strain, yield strength, and fracture strength are listed in Table 3. It can be seen that both alloys exhibit excellent mechanical properties, indicating that the removal of expensive Co elements is feasible. Previous studies have shown that the unique morphology of FCC/B2-type EHEAs plays an important role in plastic deformation [29]. As a soft phase, plastic deformation initially occurs in the FCC phase due to dislocation multiplication and slip, and the overall fraction of the FCC phase provides the possibility of a large compression ratio [30]. Inversely, the soft FCC phase starts plastic deformation while the hard B2 phase still maintains elastic deformation, which will result in a large number of dislocations being piled up at the FCC/B2 phase boundaries. During deformation, a large number of FCC/B2 phase interfaces can hinder the movement of dislocations, thus improving the strength and strain-hardening ability of the EHEAs. With the increase of strain, the hard B2 phase undergoes plastic deformation, and the Cr-rich BCC nanoprecipitates will hinder the dislocation movement, further improving the strength of the EHEAs. The coordinated deformation of the soft FCC phase and hard B2 phase makes the two EHEAs have an outstanding combination of strength and compressibility.

### 3.3. Potentiodynamic-Polarization Studies

**Figure 7:**
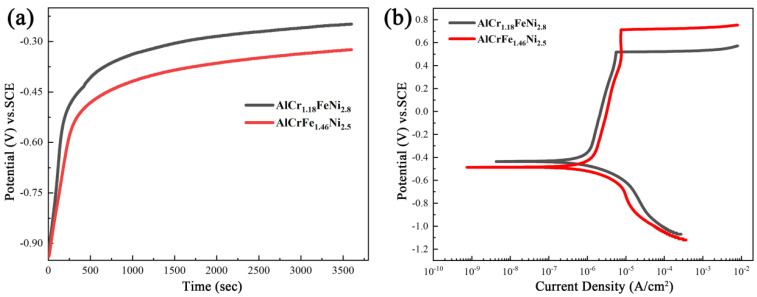
(**a**,**b**) Open circuit potential and potentiodynamic polarization curves of the AlCr_1.18_FeNi_2.8_ and AlCrFe_1.46_Ni_2.5_ alloys in 3.5 wt.% NaCl solution, respectively.

Electrochemical experiments were performed to analyze the corrosion resistance of AlCr_1.18_FeNi_2.8_ and AlCrFe_1.46_Ni_2.5_ alloys. Figure 7a shows the representative open circuit potential (OCP) curves of the samples in 3.5 wt.% NaCl solution at room temperature. With the increase of time, the open circuit gradually remains constant, indicating that the steady-state potential has been reached. Figure 7b presents the polarization curves of the two alloys in the 3.5 wt.% NaCl solution [31,32]. The direct transition from the Tafel zone stables the passive zone of the two alloys without active–passive transition. The passive film is formed spontaneously under the action of corrosion potential. To further understand the corrosion behavior of the two alloys, the relevant electrochemical parameters are listed in Table 4, including corrosion potential (E_corr_), corrosion current density (I_corr_), pitting potential (E_P_), and passive current density (I_pass_). Obviously, the AlCr_1.18_FeNi_2.8_ alloy has higher E_corr_ (−436 mV) and lower I_corr_ (7.42 μA∙cm^−2^) than that of AlCrFe_1.46_Ni_2.5_ alloy, indicating that AlCr_1.18_FeNi_2.8_ alloy has better corrosion resistance.

### 3.4. EIS Study

**Figure 8:**
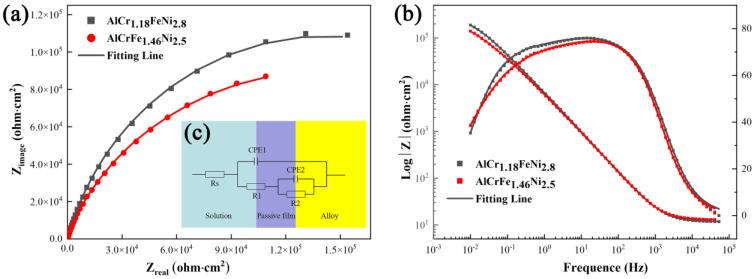
EIS results of AlCr_1.18_FeNi_2.8_ and AlCrFe_1.46_Ni_2.5_ alloys in 3.5 wt.% NaCl solution. (**a**) Nyquist plots; (**b**) Bode plots; (**c**) Equivalent electrical circuit representative of the electrode interface for the HEAs in the 3.5 wt.% NaCl solution.

In the study of corrosion and passivation processes, electrochemical impedance can be used to obtain information about the electrochemical processes on the surface of materials. Figure 8 shows the Nyquist and Bode plots of AlCr_1.18_FeNi_2.8_ and AlCrFe_1.46_Ni_2.5_ alloys. The Nyquist plots for both alloys only display a capacitor ring and semicircles arcs with centers depressed below the *x*-axis, indicating that the corrosion behavior of AlCr_1.18_FeNi_2.8_ and AlCrFe_1.46_Ni_2.5_ alloys in 3.5 wt.% NaCl solution is mainly through charge transfer rather than ion diffusion [33]. The radius of the semicircle reflects the corrosion resistance of different materials, and the larger the diameter of the semicircle, the higher the corrosion resistance of the passive film [34]. According to the Nyquist diagram, the corrosion resistance of AlCr_1.18_FeNi_2.8_ alloy is better than that of AlCrFe_1.46_Ni_2.5_ alloy, which is consistent with the results of potentiodynamic polarization. In the Bode plots, only the one-time constant is detected. However, when considering the actual reaction of the sample surface, this may be the overlap of the two constants. Additionally, the phase angle of the two alloys is close to zero in the high-frequency range. The impedance modulus value is almost constant, which indicates the two alloys have similar electrical resistance behavior. The AlCr_1.18_FeNi_2.8_ and AlCrFe_1.46_Ni_2.5_ alloys have similar impedance modulus and maximum phase angle in the middle-frequency and low-frequency range and have similar capacitive behavior [35]. The θ value of the Bode plots of AlCr_1.18_FeNi_2.8_ alloy in the mid-frequency range is greater than that of AlCrFe_1.46_Ni_2.5_ alloy, indicating that the passivation film formed in 3.5 wt.% NaCl solution is more stable than that of AlCrFe_1.46_Ni_2.5_ alloy [36]. As shown in Figure 8c, the ZSimpwin and Zview2 software are used to establish an equivalent circuit model for quantitative analysis of impedance data. The equivalent circuit consists of the solution resistance Rs, the passivation film and the capacitive behavior of the double layer CPE_1_ and CPE_2_, the passivation film resistance R_1_, and the charge transfer resistance R_2_ [37]. A constant phase element (CPE) can be replaced by pure capacitors to explain the heterogeneity of metal surfaces in the solution. The two alloys fit chi-square values of the order of magnitude less than or equal to 10^−3^ [36]. The higher the charge transfer resistance is, the lower the corrosion rate of the material is [37]. Table 5 shows the electrochemical parameters of the equivalent circuit fitting. There is no significant difference in Rs between the two alloys. The R1 value of AlCr_1.18_FeNi_2.8_ alloy is one order of magnitude higher than that of AlCrFe_1.46_Ni_2.5_ alloy, while the R2 value is slightly lower than that of AlCrFe_1.46_Ni_2.5_ alloy.

In summary, the AlCr_1.18_FeNi_2.8_ alloy shows a larger semicircle diameter in Nyquist plots and impedance modulus in Bode plots. Combined with the results of the polarization curves, the R_2_ value of AlCrFe_1.46_Ni_2.5_ alloy is slightly higher than that of AlCr_1.18_FeNi_2.8_ alloy. The passive zone of AlCrFe_1.46_Ni_2.5_ alloy is wider. However, the larger i_pass_ value of AlCrFe_1.46_Ni_2.5_ alloy indicates insufficient protection of the passive layer [38]. Overall, the superior corrosion properties of AlCr_1.18_FeNi_2.8_ alloy over AlCrFe_1.46_Ni_2.5_ alloy are attributed to the higher volume fraction of the FCC phase and the higher nickel content [37,39].

## 4. Conclusions

In summary, we establish a mathematical rule for accelerating the prediction of low-cost Co-free AlCr_a_Fe_b_Ni_c_ FCC/B2-structured eutectic medium entropy alloys. On this basis, AlCr_1.18_FeNi_2.8_ and AlCrFe_1.46_Ni_2.5_ EMEAs have been successfully designed and prepared. The microstructure, compression properties, and corrosion resistance of the two alloys are studied. The following conclusions can be drawn:

1. The mathematical rules are: c ≥ 1.0, 4.38a + 4.28b + 3.97c ≈ 20.55 and c − a ˃ 1.0. Moreover, the experiment of two alloys with lamellar eutectic structures verified the validity of mathematical rules.
2. The two alloys exhibit outstanding compressive properties, with a fracture strain > 40%, a yield strength higher than 500 MPa, and a fracture strength higher than 2450 MPa.
3. Both the alloys exhibit good corrosion resistance in 3.5 wt.% NaCl solution. Among them, the AlCr_1.18_FeNi_2.8_ EHEA exhibits better corrosion resistance due to the higher content of the FCC phase.

## Figures and Tables

**Table 1:** Physiochemical properties for the used elements (from the periodic table software).

	Al	Cr	Fe	Ni
M_i_	26.98	51.996	55.84	58.69
ρ_i_	2.7	7.15	7.86	8.908
(VEC)_i_	3	6	8	10

**Table 2:** Chemical composition of different phases in the AlCr_1.18_FeNi_2.8_ and AlCrFe_1.46_Ni_2.5_ alloys by EDS (at. %).

Alloy	Phase	Element (at. %)			
		Al	Cr	Fe	Ni
AlCr_1.18_FeNi_2.8_	Nominal	16.72	19.73	16.72	46.82
	B2	34.56	7.70	8.76	48.97
	FCC	13.17	24.10	19.67	43.06
AlCrFe_1.46_Ni_2.5_	Nominal	16.98	16.98	24.50	41.95
	B2	22.72	7.72	13.34	56.21
	FCC	8.57	23.54	32.86	35.03

**Table 3:** Compressive properties of AlCr_1.18_FeNi_2.8_ and AlCrFe_1.46_Ni_2.5_ alloys.

Alloys	Yield Strength (MPa)	Fracture Strength (MPa)	Fracture Strain (%)
AlCr_1.18_FeNi_2.8_	516	2462	40.1
AlCrFe_1.46_Ni_2.5_	550	2551	45.4

**Table 4:** Electrochemical parameters of AlCr_1.18_FeNi_2.8_ and AlCrFe_1.46_Ni_2.5_ alloys specimens in 3.5 wt.% NaCl solution.

Alloys	E_corr_ (mV_SCE_)	I_corr_ (μA∙cm^−2^)	E_P_ (mV_SCE_)	I_pass_ (μA∙cm^−2^)
AlCr_1.18_FeNi_2.8_	−436	7.42	519	5.57
AlCrFe_1.46_Ni_2.5_	−487	9.25	713	7.49

**Table 5:** Equivalent circuit parameters for impedance spectra of AlCr_1.18_FeNi_2.8_ and AlCrFe_1.46_Ni_2.5_ alloys in 3.5 wt.% NaCl solution.

Alloys	R_s_ (Ω⋅cm^2^)	R_1_ (kΩ⋅cm^2^)	R_2_ (MΩ⋅cm^2^)	CPE_1_ (×10^−5^ Ω^−1^⋅cm^2^⋅S^n^)	n_1_	CPE_2_ (×10^−5^ Ω^−1^⋅cm^2^⋅S^n^)	n_2_	χ^2^
AlCr_1.18_FeNi_2.8_	11.71	45.13	0.24	2.86	0.86	62.48	0.70	0.3 × 10^−3^
AlCrFe_1.46_Ni_2.5_	12.91	5.05	0.30	2.76	0.87	1.29	0.54	0.1 × 10^−3^
